# Prediction of severe infections in chronic lymphocytic leukemia: a simple risk score to stratify patients at diagnosis

**DOI:** 10.1007/s00277-024-05625-y

**Published:** 2024-01-18

**Authors:** Roberta Murru, Andrea Galitzia, Luca Barabino, Roberta Presicci, Giorgio La Nasa, Giovanni Caocci

**Affiliations:** 1 Hematology and Stem Cell Transplantation Unit, Ospedale Oncologico A. Businco, ARNAS G. Brotzu, Cagliari, Italy; 2 Department of Medical Sciences and Public Health, University of Cagliari, Cagliari, Italy

**Keywords:** Chronic lymphocytic leukemia, Infection, Hypogammaglobulinemia, Risk score, Immune deficiency

## Abstract

Chronic Lymphocytic Leukemia (CLL) is well-known for increasing susceptibility to infections. Factors such as immune dysregulation, IGHV status, hypogammaglobulinemia, and patient comorbidity and treatment, contribute to higher infection rates and mortality. However, the impact of hypogammaglobulinemia on infection rates is controversial. We aimed to identify clinical and biological parameters linked to the risk of severe infectious events. Additionally, we set up a straightforward risk infection score to stratify CLL patients at diagnosis, thereby enabling the development of suitable infection prevention strategies. We retrospectively evaluated 210 unselected CLL patients diagnosed between 1988 and 2018. This evaluation encompassed demographics, Binet stage, immunoglobulin (Ig) levels, treatment history, comorbidities, and IGHV mutational status at diagnosis. The frequency and severity of infectious events were recorded. Analysis revealed that age, IGHV mutational status, Binet stage, and hypogammaglobulinemia were statistically associated with the Time to First Infection (TTFI) in univariate and multivariate analyses. Using hazard ratios from the multivariate analysis, we finally devised a risk scoring system that integrated age, IGHV mutational status, immunoglobulin levels, and Binet stage to stratify patients at diagnosis based on their specific infection risk. In our cohort, disease progression and infections were the leading cause of death. These findings pointed out the clinical need for a screening process strategic for defining infectious risk at the time of CLL diagnosis, with a significant enhancement in the clinical management of these patients.

## Introduction

In CLL patients, infectious complications emerge as a significant contributor to morbidity and mortality [1]. It is estimated that approximately 80% of CLL patients experience a severe infectious event throughout the disease [2, 3]. The mortality rate due to infections ranges from 30 to 50% [4, 5]. Moreover, patients with a history of infections within the first year after diagnosis have exhibited lower overall survival [6, 7]. Furthermore, patients with CLL demonstrated heightened susceptibility to SARS-CoV-2 infections during the COVID-19 pandemic due to their intrinsic state of immunosuppression [8, 9]. Moreover, immune dysfunction associated with CLL results in ineffective serological response to vaccines against COVID-19 [10], even after boosting dose [11, 12].

The underpinning pathogenesis of this increased risk of infection in CLL patients is multifactorial,

involving intrinsic immune dysregulation, which predominantly affects the humoral immune system, advanced disease stage, and patient-related factors such as advanced age and comorbidities. Additionally, treatment-induced immunosuppression compounds this vulnerability [2, 3, 13, 14].

Hypogammaglobulinemia is the most frequently described immune disorder among CLL patients, occurring with a frequency ranging from 20 to 70% of cases, depending on the heterogeneity of the populations analyzed and the immunoglobulin class considered [15–20]. Despite its prominence, the impact of hypogammaglobulinemia on infection rates remains a controversial topic [14]. Several groups have reported an association between hypogammaglobulinemia and severe infections [17, 18, 20–23], but others suggested that immunoglobulin deficiency does not specifically correlate with the occurrence of infections [16, 24, 25].

Unmutated IGHV status has been reported to be associated with a higher incidence of infections in CLL, even in patients without hypogammaglobulinemia [20–22, 25].

Although factors associated with increased risk of infection have been well described in the literature [2, 13, 14, 26, 27], current guidelines lack clear indications on patient stratification [28]. To this end, Agius and colleagues developed a machine learning model capable of identifying patients at risk of infection within 2 years of CLL diagnosis [29]. Moreover, Mauro and colleagues developed a scoring system to assess the risk of infection in CLL patients treated with ibrutinib and/or rituximab [30].

This study aimed to define clinical and biological parameters associated with the risk of developing severe infectious events. Additionally, we devised an easy-to-use infectious risk score to facilitate stratification of CLL patients in clinical practice at diagnosis.

## Materials and methods

We retrospectively evaluated 210 unselected CLL patients diagnosed between 1988 and 2018 according to international workshop on CLL (iwCLL) guidelines [31]. Patient data were obtained from medical records, including demographics, treatment history, Binet stage at the time of diagnosis, IGHV mutational status, immunoglobulin levels (IgG, IgA, and IgM) at diagnosis, comorbidities assessed through the Cumulative Illness Rating Scale (CIRS) and occurrence of secondary tumors. TP53 aberrations and FISH abnormalities were not included for the analysis because the majority of patients did not undergo this evaluation at diagnosis, as recommended by guidelines [31]. Moreover, unlike IGHV mutational status, FISH abnormalities and TP53 can change over time through a process of clonal evolution. Immunoglobulin levels were categorized as low when IgG was below 700 mg/dl, IgA below 70 mg/dl, and IgM below 40 mg/dl. Simultaneous deficits in two or more Ig types were designated as combined antibody deficiency (CAD). Severe infectious events (graded ≥ 3) that occurred from diagnosis to the end of follow-up were included with event severity assessed by the Common Terminology Criteria for Adverse Events (CTCAE), version 5.0.

The primary aim was to evaluate the Time to First Infection (TTFI), while secondary ones were to assess the incidence of infection per 100 patient-year and the overall survival (OS).

TTFI was calculated from the diagnosis date to the occurrence of the first infection, the last follow-up (censored), or death (censored). OS was calculated from the diagnosis date to death from any cause or the last follow-up (censored). Survival analyses were executed using the Kaplan-Meier method, with differences in survival compared via the log-rank test in univariate analysis and Cox’s proportional hazard regression model in multivariate analysis to ascertain Hazard Ratios (HR). Categorical variables were compared with the Chi-square test, Fisher’s exact test, and a binary logistic regression model. Continuous variables underwent comparison using the Mann-Whitney or Wilcoxon signed-rank tests for unpaired and paired data, respectively.

*P* values below 0.05 were deemed statistically significant. The status of all included patients was updated on July 20, 2022, and the follow-up was concluded after 25 years.

Based on the findings from the multivariate analysis, we devised a scoring system to predict TTFI. The scoring system underwent internal validation by applying the bootstrap method, defining the number of bootstrap samples as 100. To evaluate its predictive capability, the area under the curve receiver operating characteristic curve (AUC) was computed, accompanied by 95% confidence intervals (CIs) for statistical significance. The statistical “R” software was used to perform all the analyses and plot figures.

## Results

### Patients’ characteristics

The median age at diagnosis was 64 years (range: 37–88). The median follow-up time was 11.03 years (range: 0.19–25). The median CIRS was 4 (range: 0–12). Among the 210 analyzed patients, 76 were female and 134 male (M:F ratio, 1.8:1). According to the Binet stage, 141 were in stage A, while 43 and 26 were in stages B and C, respectively. IGHV mutational status was available for 134 patients: 88 had a mutated status, and 46 had an unmutated status. 60% (126) of patients received at least one line of therapy. During their therapeutic history, 45 received fludarabine-based therapy, 37 alkylating agents, 77 Monoclonal Antibodies (MoAb) anti-CD20 (mainly rituximab), while 20 patients received targeted therapy (BTKi, PI3Kδi, BCL2i). All patients were treated according to iwCLL criteria, and none received CLL-directed treatment due to an infection. Fifty patients had low IgG levels, 50 had low IgA, and 72 had low IgM. Among them, 51 patients had at least one Ig class deficit, 34 had a two-class deficit, and 19 had a three-class deficit. Additionally, 53 patients had CAD (Table 1).

**Table 1:** Patients’ characteristics and demographics. CIRS: Cumulative Illness Rating Scale; CAD: Combined Antibody Deficiency; N° Ig: number of immunoglobulin class; MoAb: Monoclonal Antibodies

	All *N* = 210	Infections *N* = 70	No Infections *N* = 140	*p*-values
SEX				0.8
F	76	24	52	
M	134	46	88	
Age				0.004
> 65 years	97	43	55	
< 65 years	113	27	85	
Binet stage				0.001
A	141	40	101	
B	43	13	30	
C	26	17	9	
CIRS				0.045
≥ 6	50	23	27	
< 6	160	47	113	
IgG (*N* = 185)				0.025
> 700 mg/dl	135	44	91	
< 700 mg/dl	50	26	24	
IgA (*N* = 182)				0.032
> 70 mg/dl	132	40	88	
< 70 mg/dl	50	26	24	
IgM (*N* = 182)				0.48
> 40 mg/dl	106	38	68	
< 40 mg/dl	76	32	44	
N°Ig deficit (*N* = 182)				0.049
0	106	26	54	
1	51	16	33	
2	34	16	18	
3	19	12	7	
CAD (*N* = 182)				0.02
Absent	129	42	87	
Present	53	28	25	
IGHV (*N* = 134)				0.0002
Mutated	88	21	67	
Unmutated	46	24	22	
Treatment Status				< 0.0001
untreated	81	12	69	
treated	129	54	75	
Type of 1st Therapy				0.17
Rituximab-Bendamustine	18	9	9	
Fludara-based	45	14	31	
MoAb-based	22	8	14	
Other	44	23	21	
Target Therapy				0.22
no	100	39	61	
yes	29	15	14	

IgA levels were significantly lower in Binet stages B and C [mean 178 mg/dl (95% CI 153–202) versus 120 mg/dl (95% CI 90–149) versus 111 mg/dl (95% CI 66–156); *p* = 0.002], and in patients with unmutated IGHV [mean 174 mg/dl (95% CI 146–201) versus 128 mg/dl (95% CI 92–163); *p* = 0.006].

### Incidence of infections and time to first infection

During the disease course, 70 patients (33%) reported at least one severe infectious event (grade ≥ 3 CTCAE) requiring hospitalization and intravenous antimicrobial drug administration. The most common infections were pneumonia (17% of patients), skin and soft tissue infections (7%), sepsis (7%), and meningoencephalitis (2%). Bacteria were the most common pathogen and, among these, Gram-negative were more frequent than Gram-positive.

Patients who experienced an infection in the first 12 months after diagnosis had significantly lower IgG and IgA levels [mean 998 mg/dl (95% CI 914–1081) versus 753 mg/dl (95% CI 735–971), *p* = 0.03 and 159 (95% CI 140–177) versus 119 mg/dl (95% CI 14–223), *p* = 0.03, respectively].

The incidence rate of severe infections per 100 patient-years was 4.43 (95% CI 3.66–5.32). Patients over 65 years had a higher rate of 6.55 per 100 patient-years (95% CI 5.08–8.29) compared to 3.06 (95% CI 2.25–4.06) in younger patients (*p* < 0.0001). Treated patients had a rate of 6.64 (95% CI 5.41–8.06) compared to 1.43 (95% CI 0.8–2.34) in untreated patients (*p* < 0.0001). Binet stage C patients had a significantly higher rate of 17.83 (95% CI 12.88–23.73) compared to 4.46 (95% CI 2.66–6.69) for B stage and 2.94 (95% CI 2.22–3.81) for A stage (*p* < 0.0001). Patients with low IgG levels had a rate of 7.56 (95% CI 5.32–10.36) compared to 4.29 (95% CI 3.39–5.35) in those with normal IgG levels (*p* = 0.005). Patients with low IgA levels had a rate of 8.56 (95% CI 6.21–11.44) compared to 4.05 (95% CI 3.17–5.1) in those with normal IgA levels (*p* = 0.0001). Patients with low IgM levels had a rate of 7.15 (95% CI 5.46–9.17) compared to 3.83 (95% CI 2.88–4.98) in those with normal IgM levels (*p* = 0.001). Patients with CAD had a rate of 8.77 (95% CI 6.39–11.67) compared to 4 (95% CI 3.12–5.04) in those without CAD (*p* < 0.0001). Patients with unmutated IGHV had a rate of 8.63 (95% CI 6.05–11.85) compared to 2.65 (95% CI 1.82–3.72) in those with mutated IGHV (*p* < 0.0001). Patients with a CIRS score of ≥ 6 had a rate of 6.9 (95% CI 5-9.24) compared to 3.66 (95% CI 2.86–4.61) in those with a lower CIRS score (*p* = 0.001).

The median TTFI for the entire cohort was not reached at 25 years. Patients older than 65 had a shorter TTFI with a median of 15.4 years [95% CI 7.91 – not reached (nr)] versus a median not reached with an HR of 2.46 (95% CI 1.5-4.0, *p* = 0.0003). CIRS ≥ 6 had a negative impact on TTFI, with a median of 14.2 years (95% CI 9.84 – nr) versus. a median not reached [HR 1.72 (95% CI 1.04–2.84), *p* = 0.035]. According to the Binet stage, patients in stage C had a significantly shorter TTFI with a median of 6.08 years (95% CI 3.66–8.59) compared to a median not reached at 25 years for both B and A stages [HR C vs. B/A 3.71 (95% CI 2.09–6.5), *p* < 0.0001]. The presence of unmutated IGHV significantly affected TTFI with a median of 12.86 years (95% CI 5.1-15.04) versus not reached [HR 4.03 (95% CI 1.9–8.6), *p* < 0.0001].

**Fig. 1:**
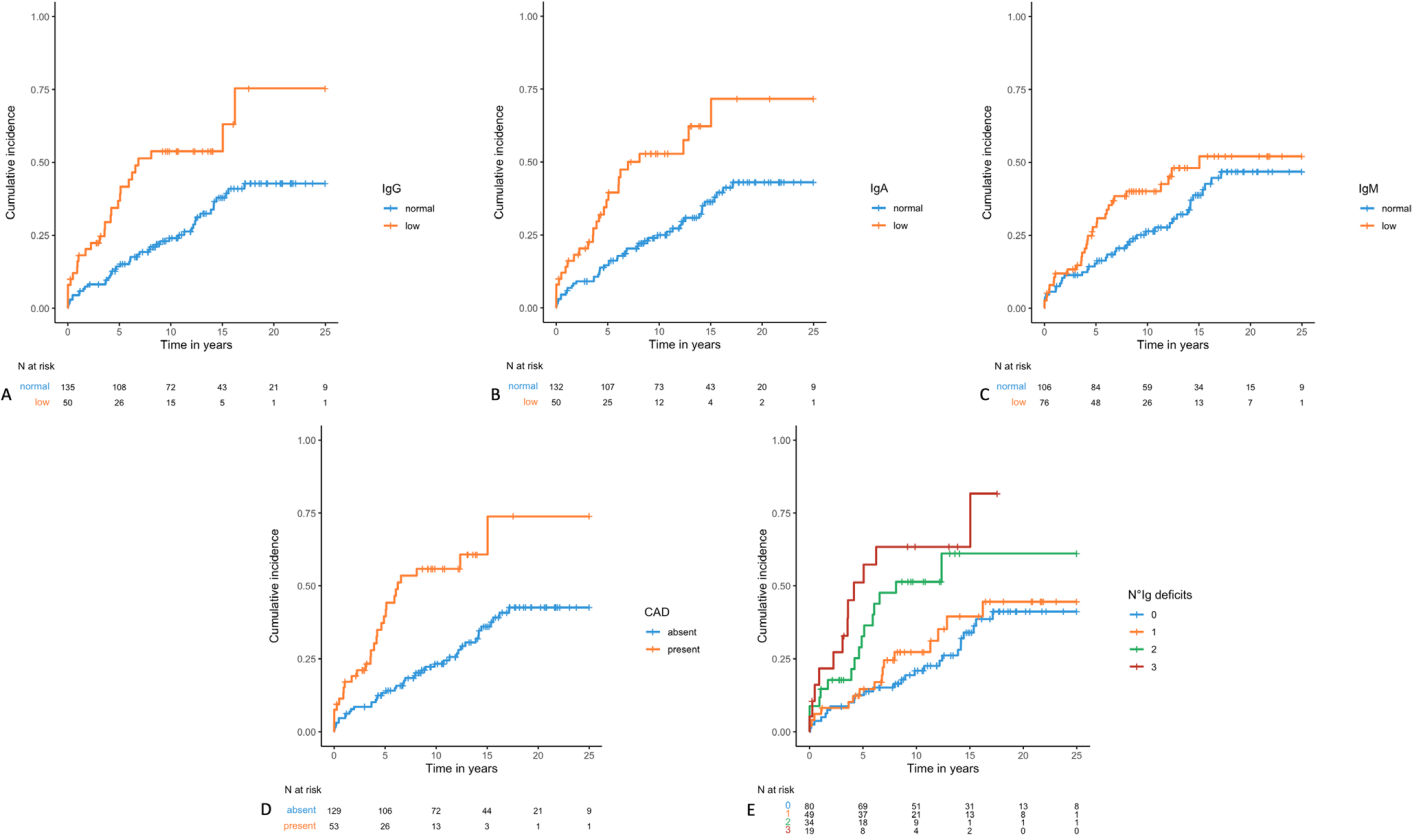
Kaplan-Meier TTFI curves according to Immunoglobulin class deficits: IgG (Panel A, *p* = 0.0003), IgA (Panel B, *p* < 0.0001), IgM (panel C, *p* = 0.04 ), CAD (panel D, *p* < 0.0001), number of Ig class deficits (panel E, *p* < 0.0001)). The x-axis represents the observation time in year while the y-axis represents the cumulative incidence of infections. CAD: Combined Antibody Deficiency

Patients with low Ig levels generally had a lower TTFI. Specifically, patients with low IgG had a median TTFI of 6.85 years (95% CI 5.07 – nr) versus nr [HR 2.55 (95% CI 1.54–4.22), *p* = 0.0003]; patients with low IgA had a median TTFI of 6.97 years (95% CI 5.07-nr) versus nr [HR 2.78 (95% CI 1.68–4.59), *p* < 0.0001]; patients with low IgM had a median TTFI of 15.04 (95% CI 7.96 – nr) versus nr [HR 1.65 (95% CI 1.01–2.73), *p* = 0.04]. Moreover, concerning the number of Ig class deficits, patients with only one deficit had a similar TTFI to patients with absence of hypogammaglobulinemia or an unknown status (median not reached in both groups). In contrast, patients with 2 or 3 Ig class deficits had a shorter TTFI: 8.1 years (95% CI 4.88 – not reached) and 4.2 years (95% CI 2.24–15.04), respectively, with a log-rank *p* < 0.0001. Taken together, patients with CAD had a TTFI of 6.24 years (95% CI 4.88 – nr) versus nr [HR 3.77 (95% CI 2.29–6.21), *p* < 0.0001] (Fig. 1).

Patients who underwent treatment had a significantly shorter TTFI with a median of 14.4 years (95% CI 12.04 – nr) compared to those not undergoing treatment, where it was not reached [HR 3.28 (95% CI 1.79–6.02), *p* < 0.001]. No differences were found between treatment regimens at the first line.

Variables associated with shorter TTFI and *p*-value < 0.05 were included in the multivariate analysis: treatment status, age > 65, CIRS ≥ 6, IGHV mutational status, Binet stage, and CAD. In this model, treatment status and CIRS ≥ 6 did not achieve statistical significance, while the other variables retained their significance: patients over 65 years had an HR of 2.4 (95% CI 1.4–4.09, *p* = 0.001); unmutated IGHV status had an HR of 3.03 (95% CI 1.52–6.06, *p* = 0.001); CAD had an HR of 3.0 (95% CI 1.76–5.12, *p* < 0.0001); Binet stage C had an HR of 3.44 (95% CI 1.8–6.55, *p* = 0.0002) (Table 2).

**Table 2:** Summary of predictive factors for TTFI in univariate and multivariate analysis and in bootstrap analysis, including HRs and respective *p*-values. ns: not significant

	UNIVARIATE			MULTIVARIATE		Bootstrap
	HR (95%CI)	*p*-value		HR (95%CI)	*p*-value	HR (95%CI)
Age > 65	2.46 (1.5–4.04)	0.0003		2.4 (1.4–4.09)	0.001	2.49 (1.59–4.56)
IgA < 70	2.78 (1.68–4.59)	< 0.0001		3 (1.76–5.12)	< 0.0001	2.83 (1.51–6.04)
IgG < 700	2.55 (1.54–4.22)	0.0003				
IgM < 40	1.65 (1.01–2.73)	0.04				
Binet C	3.71 (2.09–6.05)	< 0.0001		3.44 (1.8–6.55)	0.0002	3.74 (2.13–6.8)
IGHV unmutated	4.03 (1.9–8.6)	< 0.0001		3.03 (1.52–6.06)	0.001	3.72 (1.79–6.98)
CIRS ≥ 6	1.72 (1.04–2.84)	0.035		-	ns	-
Treatment	3.28 (1.79–6.02)	0.0001		-	ns	-

### Score to predict TTFI

**Fig. 2:**
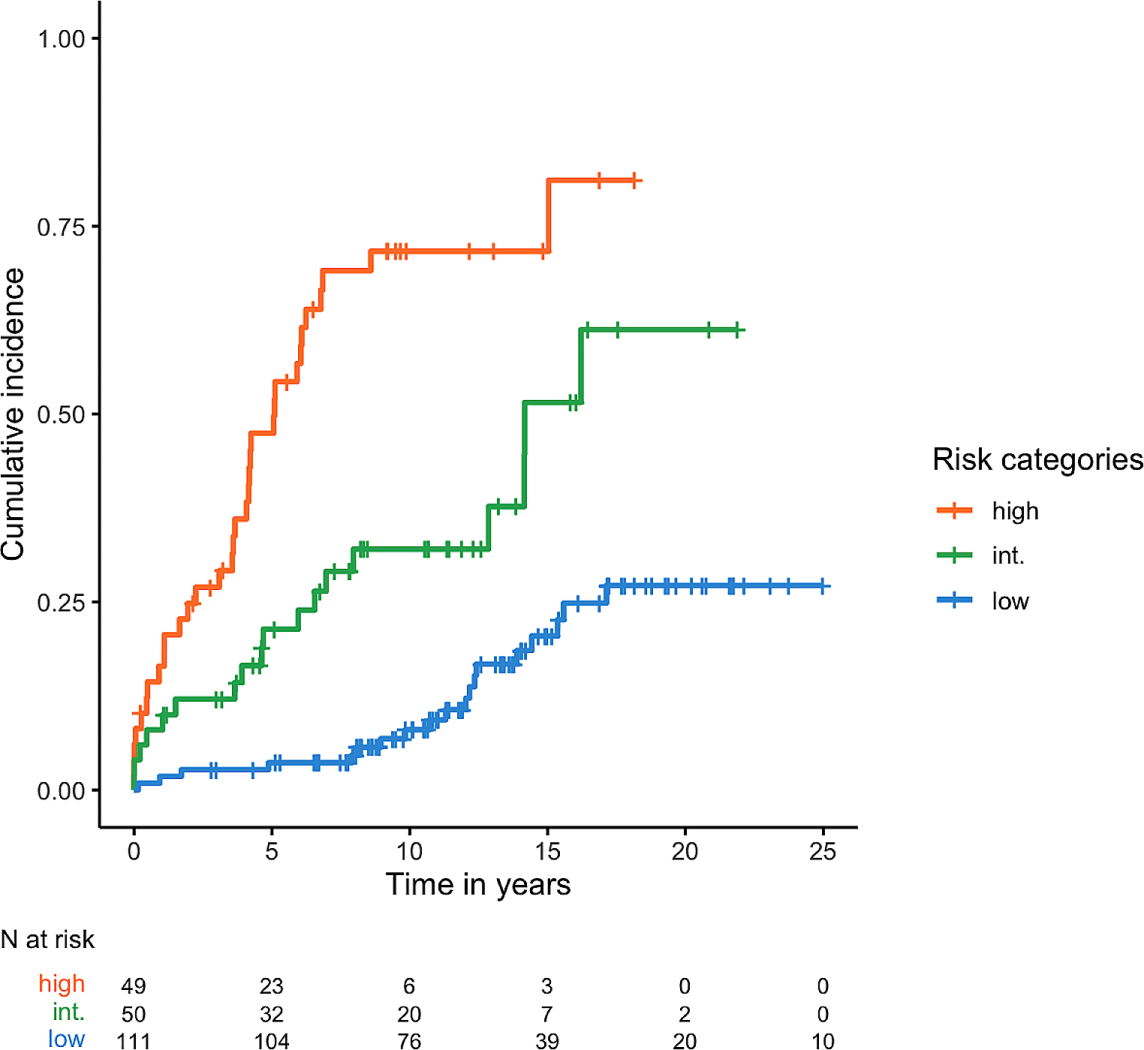
Kaplan-Meier TTFI curve according to scoring system risk categories (*p* < 0.0001)

Based on the results of Kaplan-Meier survival analysis and multivariate analysis, a scoring system was established as follows: age > 65 (2 points), unmutated IGHV status (3 points), low IgG (1 point), low IgA (1 point), low IgM (1 point), and Binet stage C (3 points). The AUC for the score to predict severe infections was 0.749 (95% CI 0.677–0.821), with the bootstrap method confirming it at 0.747 (95% CI 0.670–0.813). Patients were categorized as low risk (score 0–2), intermediate risk (score 3–4), and high risk (score ≥ 5) (Table 3). The 25-year TTFI for these risk categories demonstrated a median not reached for the low-risk group, 14.2 years (95% CI 12.86 – nr) for the intermediate-risk group [HR 3.1 (95% CI 1.6–6), *p* = 0.0007], and 5.11 years (95% CI 4.08–6.85) for the high-risk group [HR 8.81 (95% CI 4.87–15.92), *p* < 0.0001] (Fig. 2). The incidence rate of severe infections per 100 patient-year was 12.83 (95% CI 9.65–16.61) in the high-risk category, 6.57 (95% CI 4.62–9.03) in the intermediate-risk category, and 1.66 (95% CI 1.09–2.42) in the low-risk category (*p* < 0.0001).

**Table 3:** Scoring system predicting TTFI. The table presents the risk points assigned to each item in the scoring system on the left side. On the right side the table outlines the risk categories determined by the cumulative score obtained from the items, along with corresponding TTFI and the number of infection cases observed. nr: not reached

	Risk Points					
Age > 65	2					
IgA < 70	1		**SCORE**	**Risk categories**	**Median TTFI** **(95%CI)**	**N°infections/** **n°patients**
IgG < 700	1		0–2	Low risk	nr	19/111
IgM < 40	1		3–4	Intermediate risk	14.2 yrs (12.86 – nr)	18/50
Binet C	3		$\ge 5$	High risk	5.11 yrs (4.98–6.85)	33/49
IGVH-unmutated	3					

### Overall survival (OS)

Throughout the disease, 88 patients died. The causes of death included infection in 23 patients (12 sepsis, 4 pneumonia, 4 COVID-19, 1 HBV hepatitis), CLL progression in 25 patients (including 10 Richter’s transformations), secondary primary malignancy in 12 patients, other causes in 11 patients, and unknown causes in 17 patients.

**Fig. 3:**
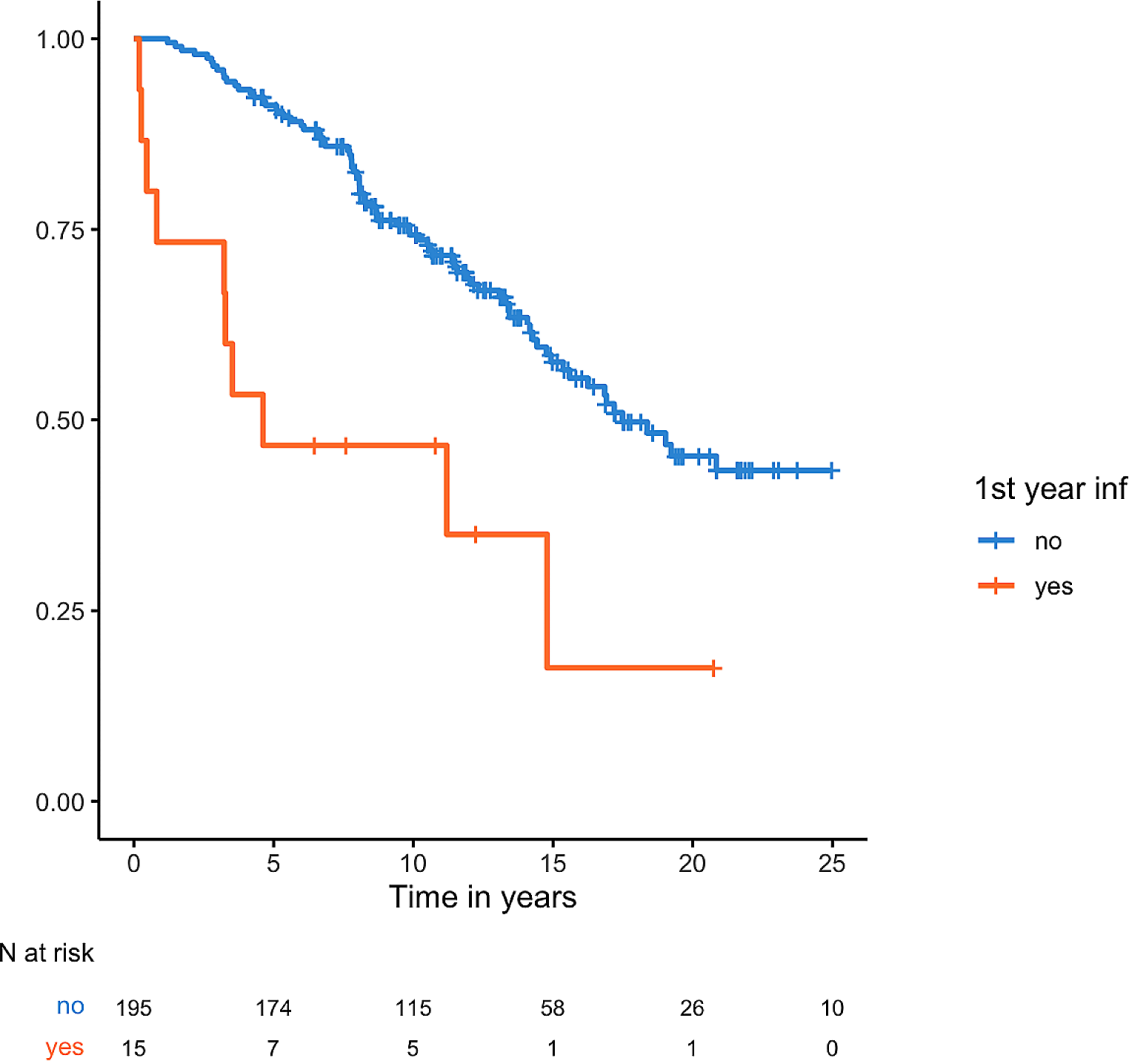
Kaplan-Meier OS curve according to history of infection within the first year from diagnosis (*p* = 0.0005)

The median OS for the entire cohort was 17.5 years (95% CI 14.4 – nr). Patients who experienced severe infections had a shorter OS, with a median of 10.51 years (95% CI 8.07–14.28) versus nr [HR 3.22 (95% CI 2.18–5.09), *p* < 0.0001]. Specifically, patients who had a severe infection in the first year after diagnosis had a median OS of 4.62 years (95% CI 3.23 – nr) versus 17.49 years (95% CI 14.93 – nr) [HR 3.06 (95% CI 1.06–8.85), *p* = 0.0005] (Fig. 3).

Regarding Ig levels, patients with IgG < 700 mg/dl had a median OS of 13.1 years (95% CI 6.67–16.85) versus 19 years (95% CI 15.36 – nr) [HR 2.06 (95% CI 1.2–3.54), *p* = 0.001]. Patients with IgA < 70 mg/dl had a median OS of 14.28 years (95% CI 8.68–19.02) versus 17.49 years (95% CI 14.43 – nr) [HR 1.67 (95% CI 0.98–2.84), *p* = 0.03]. No differences were found in patients with low levels of IgM. Patients with CAD had a median OS of 13.85 years (95% CI 6.08–16.85) versus nr[HR 2.31 (95% CI 1.45–3.68), *p* < 0.001].

Other factors associated with shorter OS were age > 65 years, with a median OS of 13.44 years (95% CI 9.39–18.85) versus nr [HR 2.45 (95% CI 1.59–3.77), *p* < 0.0001]; Binet stage C with a median OS of 8.25 years (95% CI 5.99–10.51) versus 14.27 years for B stage (95% CI 11.28-nr) versus 20.84 years for A stage (95% CI 16.25-nr), *p* < 0.0001; unmutated IGHV status with an OS of 10.5 years (95% CI 7.86 – nr) versus a median nr for mutated patients [HR 3.67 (95% CI 1.81–7.41), *p* < 0.0001]; treated patients with a median OS of 14.42 years (95% CI 11.19–18.37) versus a median nr (95% CI 16.85-nr) [HR 2.38 (95% CI 1.46–3.64), *p* = 0.0003].

Patients with CIRS ≥ 6 had a worse outcome with a median OS of 14.1 years (95% CI 10.2–18.4) versus 20.8 years (95%CI 15.6 -nr) [HR 1.74 (95% CI 1.12–2.69), *p* = 0.01].

Based on the previously identified risk categories, patients with low risk had a median OS not reached, those with intermediate risk had a median OS of 18.37 years (95% CI 10.69 – nr), and those with high risk had a median OS of 8.07 years (95% CI 5.99–13.1), *p* < 0.0001.

In the multivariate analysis, variables that independently impacted OS were Binet stage C [HR 2.99 (95% CI 1.66–5.37), *p* < 0.001], age > 65 years [HR 2.29 (95% CI 1.43–3.68), *p* < 0.001], unmutated IGHV status [HR 2.55 (95% CI 1.35–4.81), *p* = 0.003], and CAD [HR 1.72 (95% CI 1.1–2.81), *p* = 0.02]. Treatment status, infections during the first year from diagnosis, and CIRS score were not statistically associated with OS.

## Discussion

This study delves into the significance of immunoglobulin deficits as a parameter to define the infectious risk in CLL patients. While some research cohorts have suggested an association between hypogammaglobulinemia and heightened susceptibility to severe infections in CLL patients [21–23], divergent viewpoints indicate that immunoglobulin deficiency might not consistently correlate with the incidence of infections [15, 16, 24].

Agius et al. introduced a machine-learning-based model (CLL-TIM model) to predict infection risk at CLL diagnosis. Notably, their findings diverged from earlier work and failed to establish a connection between hypogammaglobulinemia and infection risk [29]. This disparity might be attributed to the lack of a standardized definition for “hypogammaglobulinemia” across different studies. Some authors address specifically IgG deficit, while others incorporate other single-class Ig deficiencies. Visentin A et al. introduced the concept of Combined Antibody deficiency (CAD) as a predictor of severe infection but also of the occurrence of secondary malignancies and even autoimmune cytopenias, highlighting it as a marker of immune failure in CLL [20, 32].

Our study supports the association between deficits across all immunoglobulin classes and the number of affected classes with infection risk. Specifically, patients exhibiting CAD were exposed to the highest risk. Remarkably, CAD maintained its significance even in the presence of other well-established infection risk factors in CLL, such as age, IGHV mutational status, and Binet stage. Notably, treatment status and CIRS score did not exhibit statistical significance in multivariate analysis.

It is reasonable to speculate that varying treatment modalities (chemoimmunotherapy, targeted therapy) influence infection risk differently. Furthermore, factors ascertainable at diagnosis hold more predictive power concerning infection-related outcomes than treatments administered at later stages of the disease.

The scoring system formulated in this study offers a rapid and easy-to-use method for evaluating infection risk in CLL patients upon diagnosis. However, its predictive utility necessitates validation through external cohorts. A noteworthy proportion (23%) of patients were classified as high-risk for infections, with nearly half of them requiring hospitalization within 5 years. A meticulous surveillance approach and contemplation of immunoglobulin replacement therapy could be warranted for this subgroup of high-risk patients.

Within our cohort, infections and CLL progression emerged as the principal causes of mortality, with individuals with a history of severe infections exhibiting substantially shortened overall survival, particularly those encountering infections within the initial year post-diagnosis. These findings resonate with the prevailing literature [6, 7]. In addition to well-established OS-associated factors (such as IGHV mutational status, Binet stage, and age), low immunoglobulin levels and CAD surfaced as predictors of OS. However, it is crucial to underscore the uncertain nature of the impact of hypogammaglobulinemia. The Mayo Clinic and Israeli CLL study groups did not find any significant association between hypogammaglobulinemia and OS [15, 33, 34]. On the other hand, Andersen et al. reported that any Ig deficiency is an adverse prognostic factor for OS [16], and, in particular, IgA deficiency seems to be a strong negative predictor of OS [18, 35]. In our cohort, CAD but not the history of infections at first year from diagnosis retained its significance on OS in multivariate analysis. This is not surprising since patients with severe infections often exhibit CAD. Therefore, CAD may reflect more aggressive disease, indicating hypogammaglobulinemia as a marker of microenvironment alteration induced by CLL and of more aggressive biological disease dynamics [14].

It must be considered that the treatment landscape for CLL has undergone significant transformation in recent years, prompting the need for reassessment of these findings in the context of target therapies. Recent evidence suggests that targeted therapy may reduce the risk of infections compared to chemotherapy [36]. Moreover, prolonged ibrutinib administration seems to partially reverse immune dysfunction by ameliorating serum IgA levels, thereby possibly reducing the incidence of infections in patients manifesting IgA improvements [37]. Recently, the E1912 trial and the combination of ibrutinib plus venetoclax have shown promising results in enhancing immune function and reducing infection susceptibility [38, 39].

Although severe and recurrent infections are currently indication for CLL-directed treatment, hypogammaglobulinemia has been shown to predict the time to first treatment [15, 18, 35]. Furthermore, improvements in stratifying the risk of infections, as well as findings of improvements in immune parameters in patients treated with novel agents, have prompted the initiation of the phase II randomized clinical trial PreVent-ACaLL. This trial aims to enhance immune function in CLL patients through early treatment intervention, employing a short 12-week course of the BTK inhibitor acalabrutinib and the BCL-2 inhibitor venetoclax in patients with high-risk features based on the CLL-TIM algorithm [29, 40]. Thus, emphasizing the importance of infection risk assessment at the time of CLL diagnosis. Our study is not devoid of limitations. Primarily, the retrospective nature of the analysis, aside from some missing data and, not least, the requirement for confirmation and validation across independent external cohorts.

## Conclusion

In conclusion, this study underscores the critical impact of infections on the survival outcomes of CLL patients. Infections, particularly within the first year following diagnosis, emerge as a determinant of overall survival, reinforcing the urgent need for vigilant infection monitoring and preventive measures in CLL management.

Furthermore, the study highlights the substantial role of hypogammaglobulinemia in mediating infection susceptibility. Even when considered alongside other firmly established risk factors, the association of CAD with infections underscores its capacity as a prognostic marker.

Considering that this study’s framework is derived from a singular institutional effort employing a retrospective analysis, our developed scoring system demonstrated its ability to anticipate adverse clinical outcomes in terms of TTFI among patients with high-risk scores for severe infectious events. Implementing a rigorous screening for infectious risk early at the time of diagnosis holds the potential to enhance the clinical management of CLL patients. This would result in more accurate adherence to therapeutic protocols and yield more favorable outcomes, particularly in the context of the recent COVID-19 pandemic and, in general, in the setting of the immunological dysregulation characterizing the disease.

Nonetheless, applying this comprehensive model warrants further exploration and validation, especially within prospective external settings. This would ensure a more robust characterization of its predictive power and the potential contribution to enhance patient care strategies in CLL management.

## Data Availability

Medical charts and databases are available at the Hematology and Stem Cell Transplantation Unit, Businco Hospital, ARNAS Brotzu, Cagliari, Italy.
